# BMP8B Increases Brown Adipose Tissue Thermogenesis through Both Central and Peripheral Actions

**DOI:** 10.1016/j.cell.2012.02.066

**Published:** 2012-05-11

**Authors:** Andrew J. Whittle, Stefania Carobbio, Luís Martins, Marc Slawik, Elayne Hondares, María Jesús Vázquez, Donald Morgan, Robert I. Csikasz, Rosalía Gallego, Sergio Rodriguez-Cuenca, Martin Dale, Samuel Virtue, Francesc Villarroya, Barbara Cannon, Kamal Rahmouni, Miguel López, Antonio Vidal-Puig

**Affiliations:** 1 Metabolic Research Laboratories, Institute of Metabolic Science, Addenbrooke's Hospital, University of Cambridge, Cambridge CB2 0QQ, UK; 2 Department of Physiology, School of Medicine-CIMUS, University of Santiago de Compostela-Instituto de Investigación Sanitaria, Santiago de Compostela 15782, Spain; 3 CIBER Fisiopatología de la Obesidad y Nutrición (CIBERobn), Santiago de Compostela 15706, Spain; 4 Medizinische Klinik und Poliklinik IV, Klinikum der Universität, Munich 80336, Germany; 5 Department of Biochemistry and Molecular Biology and Institute of Biomedicine of the University of Barcelona (IBUB), University of Barcelona, Barcelona 08028, Spain; 6 Department of Pharmacology, University of Iowa, Iowa City, IA 52242, USA; 7 Department of Internal Medicine, University of Iowa, Iowa City, IA 52242, USA; 8 The Wenner-Gren Institute, Stockholm University, Stockholm SE-106 91, Sweden; 9 Department of Morphological Sciences, School of Medicine, University of Santiago de Compostela, Santiago de Compostela 15782, Spain; 10 The Royal Veterinary College, London NW1 0TU, UK

## Abstract

Thermogenesis in brown adipose tissue (BAT) is fundamental to energy balance and is also relevant for humans. Bone morphogenetic proteins (BMPs) regulate adipogenesis, and, here, we describe a role for BMP8B in the direct regulation of thermogenesis. BMP8B is induced by nutritional and thermogenic factors in mature BAT, increasing the response to noradrenaline through enhanced p38MAPK/CREB signaling and increased lipase activity. *Bmp8b^−/−^* mice exhibit impaired thermogenesis and reduced metabolic rate, causing weight gain despite hypophagia. BMP8B is also expressed in the hypothalamus, and *Bmp8b^−/−^* mice display altered neuropeptide levels and reduced phosphorylation of AMP-activated protein kinase (AMPK), indicating an anorexigenic state. Central BMP8B treatment increased sympathetic activation of BAT, dependent on the status of AMPK in key hypothalamic nuclei. Our results indicate that BMP8B is a thermogenic protein that regulates energy balance in partnership with hypothalamic AMPK. BMP8B may offer a mechanism to specifically increase energy dissipation by BAT.

## Graphical abstract

**Graphical Abstract:**

**Figure:**
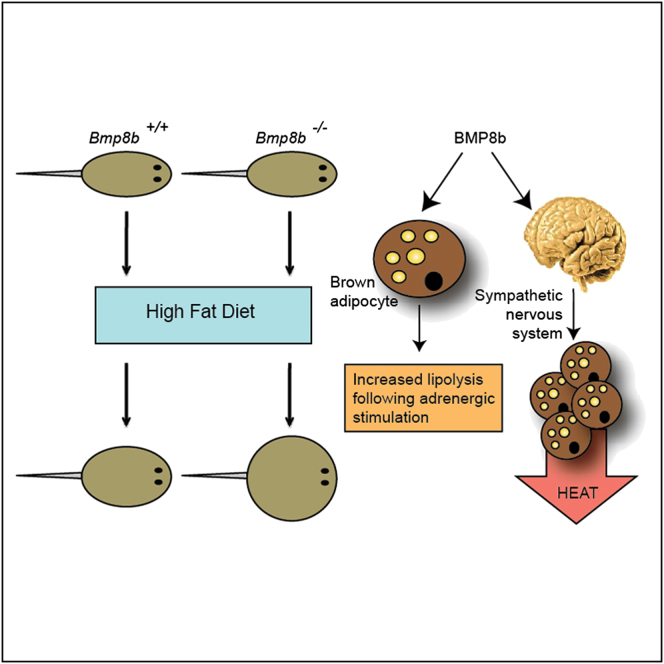

**Highlights:**

► BMP8B is expressed in BAT and is regulated in response to thermogenic stimuli ► BMP8B increases the thermogenic response of BAT to adrenergic stimulation ► Loss of BMP8B substantially increases susceptibility to diet-induced obesity ► BMP8B acts in the CNS to increase SNS activation of thermogenesis

## Teaser

Cold exposure and a high-fat diet trigger BMP8 signaling in brown adipose tissue and in the brain to boost energy expenditure.

## Introduction

Coordinated regulation of energy balance is fundamental for all organisms to produce the adequate metabolic response to changing environmental cues. Positive energy balance leads to obesity in humans and other mammals in which excess energy is stored as triglycerides in adipose tissue. Whereas white adipose tissue (WAT) functions as a storage depot for lipids, releasing energy between meals, brown adipose tissue (BAT) provides a mechanism for thermogenesis, dissipating energy as heat. The coordinated storing and burning of lipids is crucial for energy homeostasis, and the contribution of thermogenesis is demonstrated in mice, in which those lacking functional BAT become obese (Bachman et al., 2002; Lowell et al., 1993).

Originally thought to be present only in infants, functional BAT has now been widely documented in adult humans (Cypess et al., 2009; Saito et al., 2009; van Marken Lichtenbelt et al., 2009; Zingaretti et al., 2009), making thermogenesis a potential mechanism for combating obesity. Nonshivering thermogenesis (NST) exists primarily to defend core body temperature, but increasing the caloric intake of an animal also stimulates BAT. The extent to which thermogenesis can offset caloric excess is largely dependent on environmental temperature or the basal level of BAT activation. Thus, animals that are fed high-calorie diets at higher temperatures gain more weight due to reduced diet-induced thermogenesis (DIT) (Rothwell and Stock, 1986). The sympathetic nervous system (SNS) is the major regulator of BAT, signaling through β-adrenergic receptors (βARs). β1AR agonism mainly stimulates BAT recruitment, and the β3AR primarily activates the thermogenic machinery in mature brown adipocytes (Cannon and Nedergaard, 2004).

Innervation of BAT emanates from autonomic centers in the brain, including the ventromedial (Halvorson et al., 1990) and paraventricular (Amir, 1990; Madden and Morrison, 2009) nuclei of the hypothalamus (VMH and PVN, respectively). The VMH and arcuate nucleus (ARC) are also involved in appetite regulation (López et al., 2008; Morton et al., 2006); they respond to changes in nutritional status to maintain energy balance via autonomic, hormonal, and behavioral changes. We have shown previously that activation of AMP-activated protein kinase (AMPK) and fatty acid metabolism in the VMH acts to conserve energy globally and determines the level of central activation of BAT (López et al., 2010; Martínez de Morentin et al., 2012). The amount of thermogenesis resulting from a given environmental stimulus depends, therefore, on three key factors: (1) activation of central regulatory mechanisms, (2) sensitivity of BAT, and (3) the thermogenic machinery's capacity to generate heat.

Bone morphogenetic proteins (BMPs) are members of the transforming growth factor β (TGF-β) superfamily (Wozney et al., 1988). Subsequent research has revealed their pleiotropic effects in numerous tissues and physiological processes, in which different BMPs act on specific cell types (Chen et al., 2004). In adipose tissue, BMPs 2 and 4 are able to increase commitment of mesenchymal cells to the adipocyte lineage (Ahrens et al., 1993; Sottile and Seuwen, 2000; Tang et al., 2004) and drive preadipocytes to differentiate into mature white adipocytes in vitro (Bowers and Lane, 2007; Wang et al., 1993). Less is known about the role of BMPs in BAT, but BMP7 is known to drive preadipocytes toward a “brown” cell fate (Tseng et al., 2008).

Here, we describe a role for BMP8B as a signaling molecule regulating thermogenesis and energy balance. Using *Bmp8b^−/−^* mice, we demonstrate that BMP8B increases the peripheral response of BAT to adrenergic stimulation while acting centrally to increase sympathetic output to BAT. Our evidence suggests that BMPs not only signal to immature cells but also to fully differentiated mature adipocytes and neurons. In doing so, BMP8B acutely affects their activity, making BMP8B an important regulator of energy balance.

## Results

### BMP8B Is Expressed in Mature Brown Adipocytes and Responds to Thermogenic Stimuli

**Figure 1:**
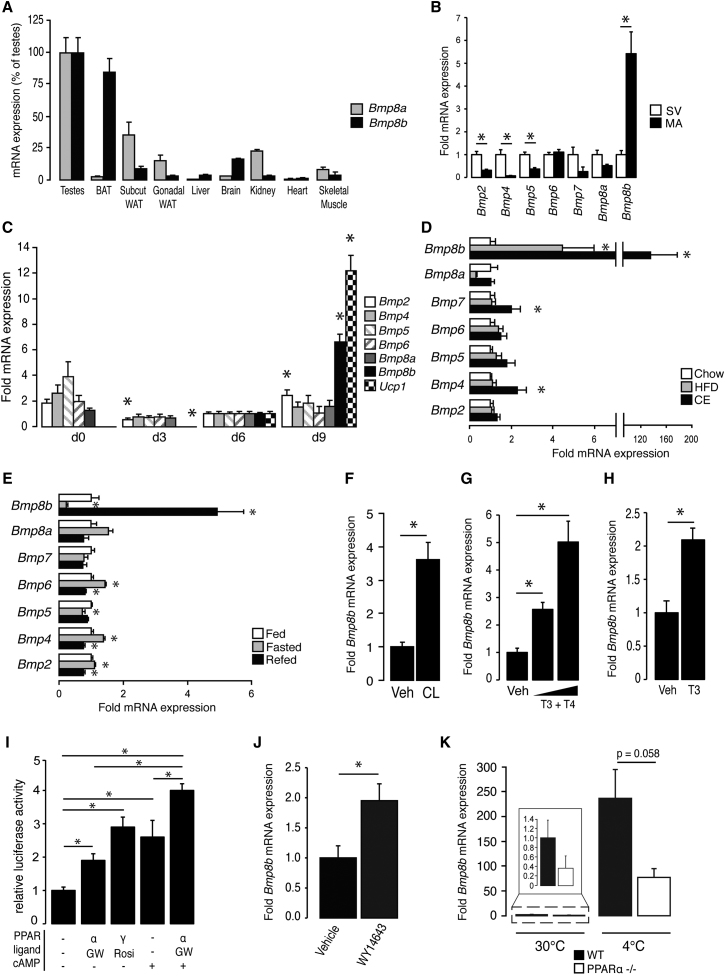
Physiological Regulation of *Bmp8b* (A) Tissue distribution of *Bmp8a* and *Bmp8b* mRNA, relative to levels in testes. n = 8 mice, age 12 weeks. (B) BMP expression in stromal-vascular (SV) and mature adipocyte (MA) fractions of BAT. n = 6, age 12 weeks. (C) mRNA expression of BMPs throughout differentiation of brown adipocytes in vitro. n = 3 experiments, each in triplicate, relative to day 6 levels. (D) BAT expression of BMPs in mice fed HFD for 12 weeks or housed at 4°C for 3 weeks (CE), relative to chow fed housed at 23°C (CHOW). n = 7, age 16 weeks. (E) BAT expression of BMPs in fed, fasted, and refed animals. (F) BAT expression of BMP8B in 12-week-old mice after 7 days of treatment with vehicle or specific β3 agonist CL 316243 (1 mg/kg/24 hr via subcut osmotic pump). (G) BAT BMP8B expression in mice given control (vehicle = 10 mM NaOH), mild (5 μg T4 + 2 μg T3/100 g/day), or intensive (20 μg T4 + 8 μg T3/100 g/day) hyperthyroid treatment. n = 3 per group, age 12 weeks. (H) *Bmp8b* expression in differentiated brown adipocytes treated with vehicle or T3 (5 nM) for 6 hr. n = 2 experiments, each in triplicate. (I) Luciferase activity in HIB-1B cells driven by *Bmp8b* promoter in response to cotransfection with PPARα or γ and specific chemical agonists Rosiglitazone or GW7647 and dibutyril-cAMP. n = 4 experiments, each in triplicate. (J) *Bmp8b* expression in mice treated with vehicle or 25 mg/kg of PPARα agonist WY14643 via five daily IP injections. (K) *Bmp8b* mRNA expression in wild-type and PPAR*^−/−^* mice at 30°C and 4°C. n = 3 per group. Error bars indicate SEM. See also Figure S1.

We examined expression levels of *Bmp8b* and its gene duplicate, *Bmp8a*, in several murine tissues, and consistent with previous findings (Zhao and Hogan, 1996), testes had the highest *Bmp8b* mRNA levels. Comparable levels were detected in BAT, with significant *Bmp8b* expression also present in the brain (Figure 1A). Perhaps not surprising given their heterologous promoters, expression patterns of *Bmp8a* and *Bmp8b* were markedly different across the tissues profiled, with *Bmp8a* almost absent from BAT and brain, yet enriched in WAT. Fractionation of BAT showed that *Bmp8b* expression was restricted to the mature adipocyte population, with levels greater than five times those in stromal-vascular cells (Figure 1B). In light of recent evidence that BMPs drive adipogenesis, we considered that BMP8B might promote differentiation but found its expression in cultured brown adipocytes to be induced only in mature cells, alongside canonical markers of thermogenic capacity (Ucp1) (Figure 1C).

Brown adipocytes in vivo display a well-defined adaptive response to sympathetic activation, which is induced by feeding a high-fat diet (HFD) or, to a greater degree, by cold exposure (Cannon and Nedergaard, 2004; Collins et al., 2004). Induction of *Bmp8b* expression was coordinated with this response in BAT and was modulated according to the strength of the stimulus, increasing 4-fold and 140-fold following HFD and cold exposure, respectively (Figure 1D). Of all the BMPs measured, BMP8B displayed the most robust response to HFD. To determine whether *Bmp8b* expression was dependent on adaptive changes to BAT, acute nutritional challenges were applied to animals in the form of 24 hr periods of fasting or refeeding. Again, *Bmp8b* expression showed the greatest magnitude of change, falling during fasting and rising dramatically after refeeding (Figure 1E).

**Figure S1:**
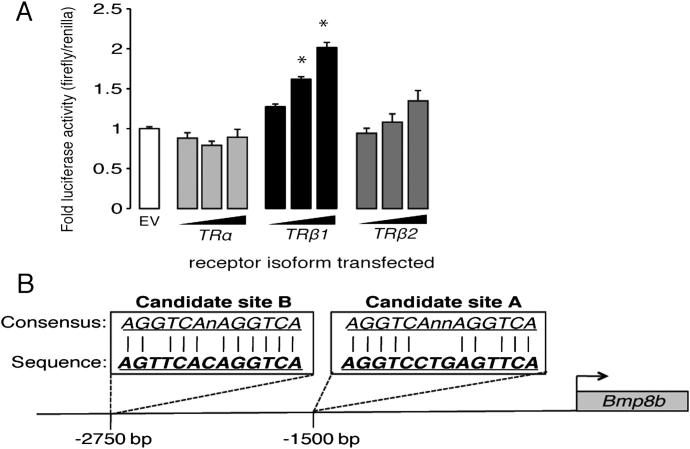
Regulation of the BMP8B Promoter, Related to Figure 1 (A) Luciferase activity in HEK293 cells driven from 3 kb of the BMP8B promoter following co-transfection with plasmids overexpressing the indicated TR isoforms. n = 3 experiments carried out in triplicate. (B) Schematic diagram of 3 kb of the murine BMP8B promoter indicating location and sequences of the 2 identified candidate PPREs. ^∗^p < 0.05 using ANOVA. Error bars indicate SEM.

Physiological activation of BAT results from increased sympathetic stimulation, with many thermogenic mechanisms responding specifically to β3-adrenergic receptor (β3AR) activation (Collins and Surwit, 2001). Activation of β3ARs by using a synthetic ligand, CL 316243, significantly increased *Bmp8b* mRNA levels in BAT in vivo (Figure 1F). Thyroid hormone, a key regulator of thermogenesis in BAT (López et al., 2010; Silva, 1995), elicited a dose-dependent increase in *Bmp8b* in mice with hyperthyroidism (Figure 1G) and brown adipocytes (treated with triiodothyronine, T3) (Figure 1H). A luciferase reporter assay demonstrated that the *Bmp8b* promoter could be driven in a dose-dependent manner by overexpression of thyroid hormone receptor β1 (TRβ1) (Figure S1A available online), the isoform required for thermogenic gene induction in BAT (Ribeiro et al., 2010).

To examine whether BMP8B lay within other known adipocyte regulatory pathways, we analyzed its promoter and identified two sites with homology to peroxisome proliferator-activated receptor (PPAR) response elements (PPREs) within ∼1,500 kb proximal to the transcription start site (Figure S1B). Overexpression of PPARα or PPARγ in BAT-derived HIB-1B cells and treatment with their agonists revealed that the *Bmp8b* promoter was responsive to both PPAR isoforms (Figure 1I). cAMP also increased transcription alone and synergistically with PPARα, the PPAR linking regulation of thermogenesis with lipid oxidation (Barbera et al., 2001). PPARα agonism also induced *Bmp8b* in BAT in vivo (Figure 1J), whereas PPARα ablation reduced *Bmp8b* expression in unstimulated and thermogenically active BAT (Figure 1K).

### *Bmp8b^−/−^* Mice Have Impaired Thermogenesis and Are Susceptible to Diet-Induced Obesity

Previous analyses found that *Bmp8b^−/−^* mice were viable and healthy but infertile due to defective primordial germ cell formation (Ying et al., 2000; Zhao and Hogan, 1996). Our initial analysis of litters from heterozygotes showed that the number of *Bmp8b^−/−^* animals obtained (11%) was significantly lower than expected Mendelian ratios. This phenotype was temperature sensitive, as *Bmp8b^−/−^* pup numbers rose to 19% after an environmental shift from 20°C to 23°C (Table S1).

**Figure 2:**
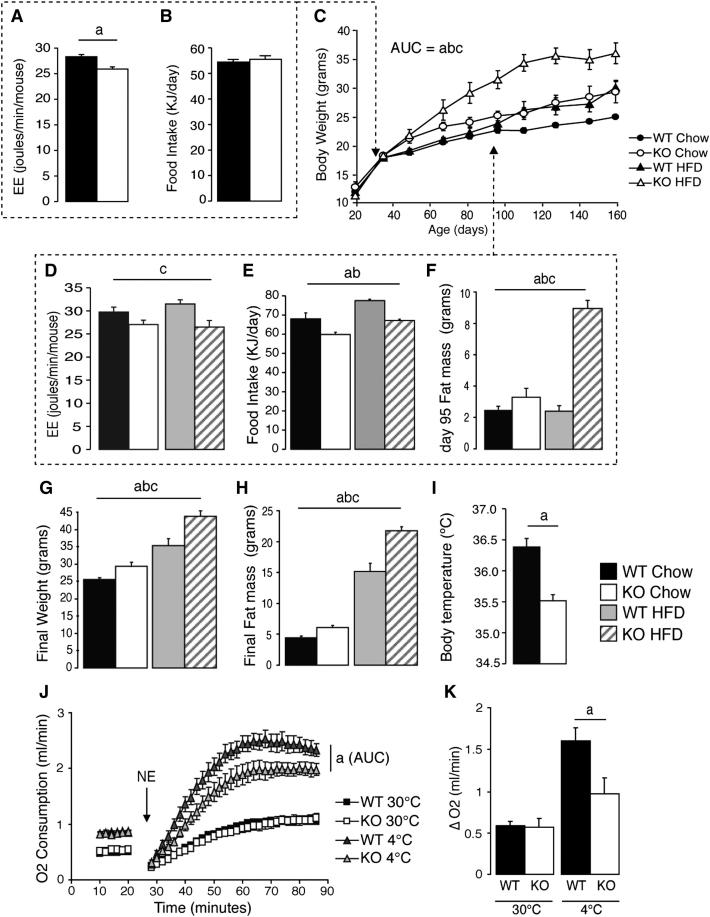
Obesogenic Phenotype of *Bmp8b^−/−^* Mice (A and B) (A) Rate of energy expenditure at 4 weeks of age by using ANCOVA (adjusted body weight = 16.98 g) and (B) food intake in the same mice. n = 6–8. (C and D) (C) Growth curves of wild-type (WT) and *Bmp8b^−/−^* (KO) animals fed chow or HFD postweaning with (D) energy expenditure by using ANCOVA (adjusted body weight = 23.82 g). (E–H) (E) Daily food intake and (F) fat mass at 95 days in the same animals, followed by (G) body weights and (H) fat mass at sacrifice (185 days). n = 7. (I) Body temperature measured via subcutaneous chip over 7 days. (J) NE stimulated oxygen consumption in WT and KO mice housed at thermoneutrality (30°C) or cold exposure (4°C) for 3 weeks, expressed as VO_2_ over time and (K) ΔVO2 from baseline. n = 7. Annotation indicates significant effect of a = genotype, b = diet, or c = significant diet-genotype interaction. p < 0.05 using ANOVA or ANCOVA. AUC, area under curve. Error bars indicate SEM. See also Figure S2 and Tables S1 and S2.

**Figure S2:**
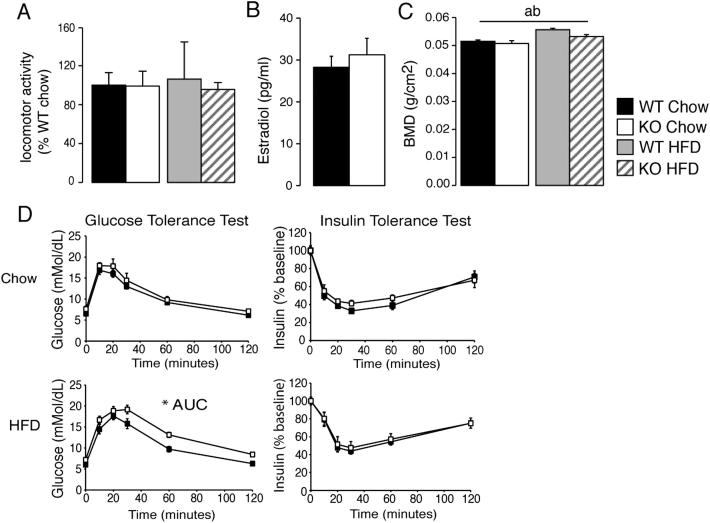
Evaluation of Metabolism in *Bmp8b^−/−^* Mice, Related to Figure 2 (A) Locomotor activity measured by beam breaks over 48 hr in mice depicted in Figure 2. (B) Serum estradiol levels in serum of chow fed animals aged 12 weeks. n = 4. (C) Bone mineral density measured by DEXA at sacrifice. n = 7. (D) Glucose tolerance test and insulin tolerance test performed in WT and KO mice fed chow and high-fat diet. Mice were given a bolus of glucose (1 g/Kg i.p) or insulin (0.75 IU/Kg i.p) corrected for body weight and subsequent measurements of blood glucose were taken at the indicated time points following injection. Error bars indicate SEM. Annotation indicates significant effect of a = genotype, b = diet or c = significant diet-genotype interaction. p < 0.05 using ANOVA, AUC = area under curve.

Postweaning, young *Bmp8b^−/−^* mice already had a reduced metabolic rate (Figure 2A) as compared to wild-type littermates with no alteration to food intake (Figure 2B). *Bmp8b^−/−^* animals subsequently displayed increased propensity for weight gain, significantly exacerbated by feeding HFD (Figure 2C). The significant reduction to metabolic rate (2 J/min) of *Bmp8b^−/−^* mice persisted throughout their weight divergence from wild-type mice and was itself exacerbated by HFD (Figure 2D). *Bmp8b^−/−^* mice consumed ∼15% less energy on chow and HFD (8 KJ/day and 10 KJ/day less, respectively) (Figure 2E) yet still became 4 g (16%) heavier on chow and 9 g (32%) heavier on HFD by the end of the study (25 weeks old) (Figure 2G). Increased body weight was accounted for by fat mass quantified at the time of calorimetry (day 95) (Figure 2F) and sacrifice (Figure 2H). Core body temperature was significantly reduced in *Bmp8b^−/−^* mice (Figure 2I), although no alterations to locomotor activity (Figure S2A), hormone levels (Figure S2B), carbohydrate metabolism (Figure S2D), or serum biochemistry (Table S2) were observed.

Assessment of maximal thermogenic capacity after adaptation to cold revealed a significant impairment in the adrenergic response of BAT of *Bmp8b^−/−^* mice, evident from a significant reduction in total oxygen consumption and reduced increase from baseline following norepinephrine (NE) injection. This was not seen in mice housed at thermoneutrality (Figures 2J and 2K).

### *Bmp8b^−/−^* Mice Display Normal BAT Expansion and Morphology but Impaired Inducibility of Thermogenic Machinery by Diet

**Figure 3:**
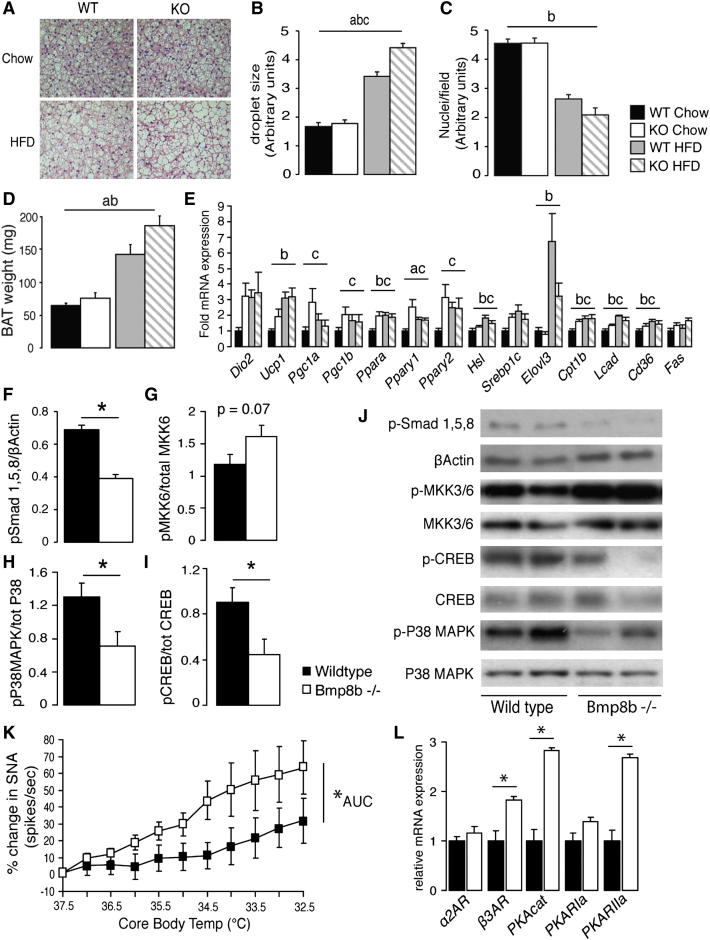
Characterization of *Bmp8b^−/−^* BAT (A) Haematoxylin and eosin sections from interscapular BAT of WT and KO animals fed chow or HFD for 4 months postweaning. (B and C) (B) Lipid droplet size and (C) number of nuclei per field, scored visually by using arbitrary units. n = 2 sections per mouse, 7 per group. (D and E) (D) Weights of the same BAT depots and (E) expression of genes associated with thermogenesis, adipogenesis, and lipid handling in BAT. n = 7 animals per group, age 5 months. (F–J) Quantification of indicated phosphoproteins in the Smad and P38 MAPK pathways in BAT of WT and KO mice with (J) representative images of blots. n = 5–8 per group. (K) Direct measurement of SNA to BAT of WT and KO mice in response to controlled lowering of body temperature for 30 min. n = 7 per group, age 12 weeks. (L) Expression of genes encoding adrenergic receptors and associated signaling proteins in BAT of WT and KO mice. n = 7 per group, age 16 weeks. Error bars indicate SEM. Annotation indicates significant effect of a = genotype, b = diet, or c = significant diet-genotype interaction, defined as p < 0.05 using two-way ANOVA. ^∗^p < 0.05 using ANOVA.

Histology revealed normal BAT morphology in *Bmp8b^−/−^* mice (Figure 3A) but larger lipid droplets following HFD (Figure 3B), indicating lower thermogenic activity (Figure 3C). BAT expansion was not impaired in *Bmp8b^−/−^* mice, which had increased BAT weight (Figure 3D) and total fat mass (Figure 2H). Thermogenesis requires induction of *Ucp1* and genes driving lipolysis, mitochondriogenesis, and β oxidation of fatty acids (Cannon and Nedergaard, 2004). BAT mRNA levels of these genes were elevated in *Bmp8b^−/−^* mice compared to wild-type mice in the basal state (chow fed) but failed to increase in response to HFD (Figure 3E). This reinforced our hypothesis that loss of BMP8B results in reduced BAT responsiveness.

BMPs signal via Smad proteins, and levels of active Smads 1, 5, and 8 were significantly reduced in *Bmp8b^−/−^* BAT (Figures 3F and 3J). P38MAPK and phospho-cAMP response element-binding protein (pCREB), essential signaling molecules for the induction of thermogenesis in BAT (Cao et al., 2001), are intrinsically linked to Smad signaling (Morikawa et al., 2009; Sellayah et al., 2011; Shibuya et al., 1998; Shim et al., 2009; Yamaguchi et al., 1999), and their levels of activation were also significantly reduced in *Bmp8b^−/−^* mice without alterations to total protein levels (Figures 3H–3J). Phosphorylation of MKK3 and MKK6, both necessary for P38MAPK activation in vitro and in vivo (Alonso et al., 2000; Remy et al., 2010), was increased in *Bmp8b^−/−^* mice (Figures 3G and 3J), suggesting that BMP8B ablation caused a blockade at this point in the adrenergic signaling cascade.

Because DIT is dependent on temperature, we asked whether its reduction in *Bmp8b^−/−^* mice was due to reduced sympathetic stimulation of BAT in response to environmental temperature. Direct recordings of sympathetic nerve activity (SNA) showed the opposite, with SNA to BAT increased in *Bmp8b^−/−^* mice compared to wild-type mice following cooling (Figure 3K). No defect in BAT reception of SNA was evident, as *Bmp8b^−/−^* mice displayed increased adrenergic membrane receptor expression in BAT, including β3AR and its downstream transducer, protein kinase A (PKA) (Figure 3L).

### BMP8B Increases the Capacity of Brown Adipocytes to Respond to Norepinephrine

**Figure 4:**
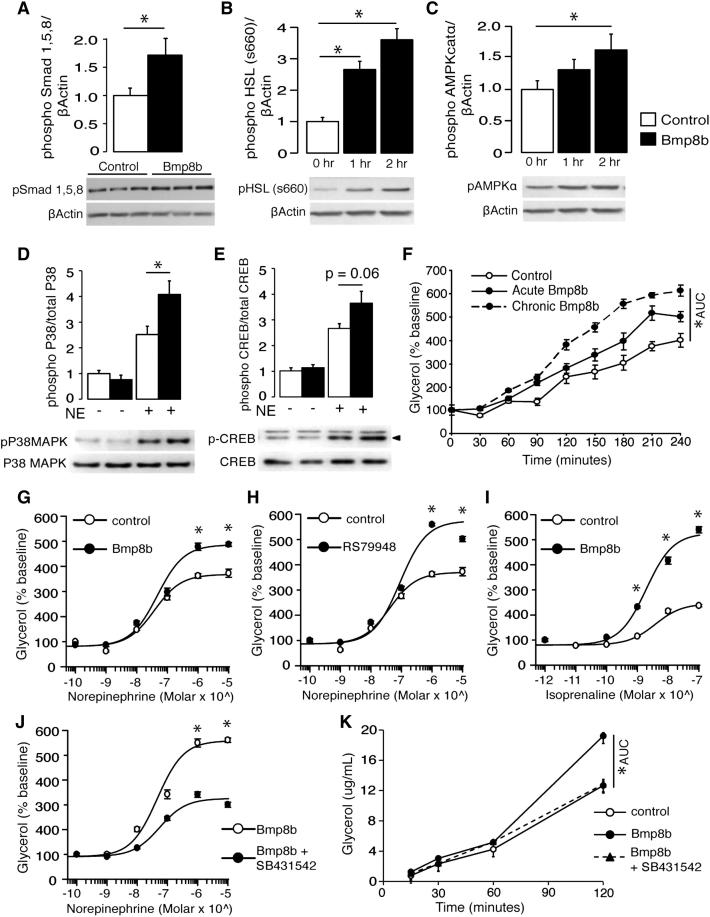
BMP8B-Mediated Alterations to Brown Adipocyte Lipolysis (A–C) (A) Levels of phospho-Smad 1/5/8 in differentiated brown adipocytes following 2 hr treatment with 100 pM BMP8B, (B) pHSL, and (C) pAMPK after 1 and 2 hr treatment. (D and E) (D) Phospho-P38 MAPK and (E) phospho-CREB levels after subsequent 10 min stimulation with NE. Blots represent three experiments performed in duplicate/triplicate with phospho levels normalized to the total protein. (F) Lipolytic activity in differentiated brown adipocytes stimulated with NE (75 nM) after treatment with vehicle (control), 100 pM BMP8B for 2 hr (acute BMP8B), or throughout differentiation (chronic BMP8B). n = 3 experiments, in triplicate. (G and H) (G) Lipolytic dose-response curves for cells pretreated for 2 hr with 100 pM BMP8B followed by 2 hr of NE and (H) NE stimulated dose-response in absence or presence of 10 μM α2AR antagonist RS79948. (I) Effect of BMP8B on isoprenaline dose response. (J) Effect of 10 μM ALK7 antagonist SB431542 on NE dose response following BMP8B treatment. (K) Effect of ALK7 inhibition on rate of NE-stimulated lipolysis in BMP8B-treated brown adipocytes. n = 8. Error bars indicate SEM. ^∗^p < 0.05 using ANOVA. AUC, area under curve. See also Figure S3.

**Figure S3:**
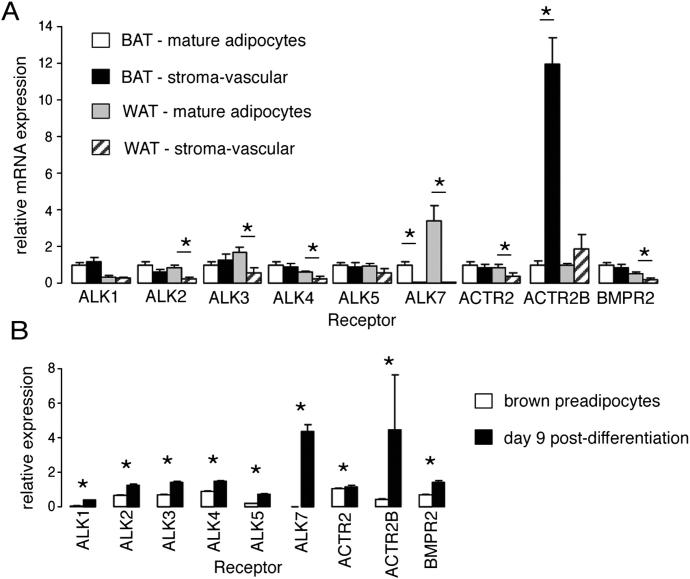
BAT Expression Profile of Candidate BMP Receptors, Related to Figure 4 (A) mRNA expression levels of potential BMP8B receptors in fractionated BAT and WAT from wild-type mice, n = 8 aged 12 weeks and (B) the same genes measured in an immortalized BAT cell line both pre- and post-differentiation. N = 3 experiments in duplicate. ^∗^p < 0.05 using ANOVA. Error bars indicate SEM.

Treatment of differentiated brown adipocytes with BMP8B resulted in increased Smad phosphorylation (Figure 4A) and activation of hormone-sensitive lipase (HSL) and AMPK (Figures 4B and 4C), which are key regulators of lipid storage and oxidation. The functional effect of BMP8B treatment was an elevated response to adrenergic stimulation, both in terms of intracellular signaling molecule activation (P38MAPK and CREB) (Figures 4D and 4E) and increased lipolytic response to NE (Figure 4F). BMP8B treatment elicited intracellular changes that “primed” cells, enabling a higher maximal lipolytic response to more potent doses of NE (Figure 4G) in a manner that resembled antagonism of the inhibitory α2 adrenergic receptors (AR) (Figure 4H). However, this effect remained even when using isoprenaline as the stimulant (which bypasses α-adrenergic receptors) (Figure 4I). Interestingly, the only BMP receptor that shares the expression profile of BMP8B in BAT is activin receptor-like kinase 7 (ALK7) (Figure S3), the antagonism of which abolished BMP8B's effect on NE-stimulated lipolysis (Figures 4J and 4K).

### Hypothalamic BMP8B Acts Centrally to Increase Sympathetic Tone to BAT

**Figure 5:**
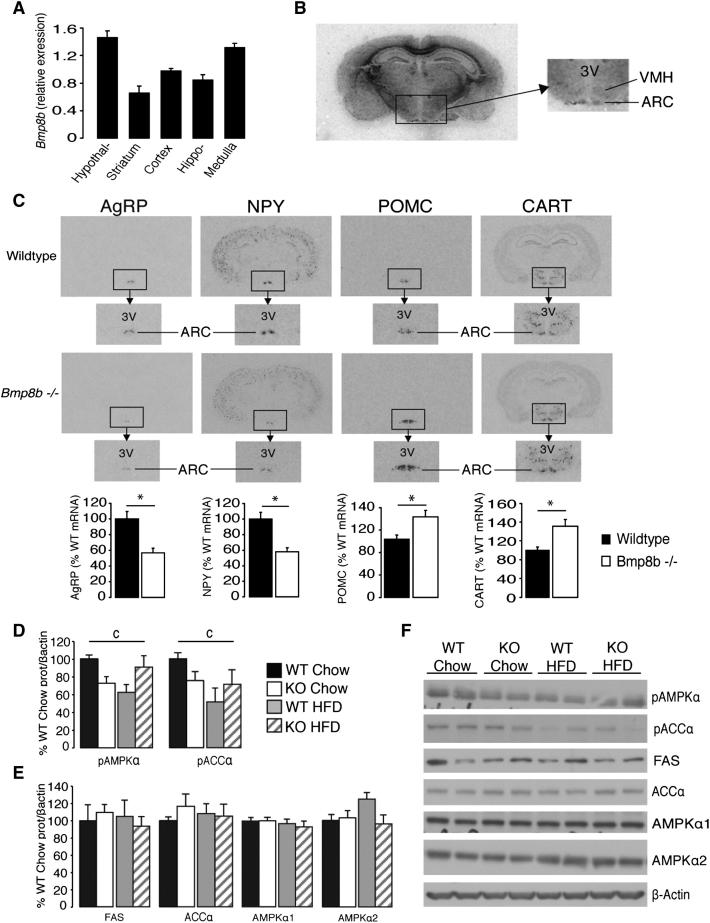
Characterization of *Bmp8b^−/−^* Brains (A) Expression of *Bmp8b* mRNA in brain regions dissected from fed mice age 12 weeks. n = 6. (B) *Bmp8b* mRNA in the ventromedial and arcuate nuclei of the hypothalamus. n = 8 mice, age 12 weeks. (C) In situ hybridization showing key neuropeptide mRNA regulating feeding (AgRP, NPY, POMC, and CART) in arcuate nucleus of the hypothalamus in WT and *Bmp8b^−/−^* mice. n = 7 per group, age 5 months, that was fed chow, accompanied by representative images and densitometric analysis. (D–F) (D) Western blot analysis of the fatty acid synthesis pathway in hypothalamus of WT and *Bmp8b^−/−^* mice following 12 weeks of chow or HFD treatment, showing levels of activated AMPKα and pACCα alongside (E) total levels of FAS, ACCα, AMPKα1, and AMPKα2, which are all normalized to β-actin with (F) representative immunoblots. n = 6–8. Error bars indicate SEM. c = significant diet-genotype interaction. p < 0.05 using ANOVA or two-way ANOVA, depending on the number of variables. See also Figure S4.

Altered sympathetic outflow to BAT and reduced food intake in *Bmp8b^−/−^* mice pointed to a possible central role of BMP8B. *Bmp8b* mRNA was present in the brain and enriched in the hypothalamus and medulla (Figure 5A). Further analysis also detected *Bmp8b* mRNA in the VMH, a nucleus long known to control thermogenesis in BAT (Halvorson et al., 1990; López et al., 2010; Woods and Stock, 1994), and in the ARC (Figure 5B), where *Bmp8b^−/−^* mice displayed a pattern of neuropeptide expression conducive to their reduced food intake (Morton et al., 2006) (Figure 5C).

**Figure S4:**
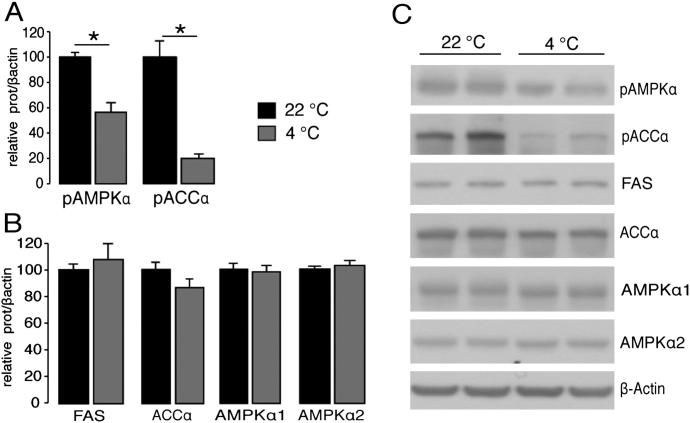
Effects of Cold Exposure on the Hypothalamic Fatty Acid Synthesis Pathway, Related to Figure 5 Western blot analysis of the fatty acid synthesis pathway in hypothalamus of wild-type and *Bmp8b^−/−^* mice following 3 weeks housing at 22°C or 4°C to induce thermogenesis in BAT, showing levels of (A) activated AMPK and activated ACCα alongside (B) levels of total FAS, ACCα, AMPKα1 and AMPKα2, normalized to β-actin with (C) representative blots, n = 8. ^∗^p < 0.05 using ANOVA. Error bars indicate SEM.

In the hypothalamus, the fatty acid synthetic pathway, which is modulated by AMPK, has been shown to be a key regulator of feeding and thermogenesis (López et al., 2008, 2010; Martínez de Morentin et al., 2012; Xue et al., 2009). In line with an overstimulation of BAT on chow diet, there was reduced activation of hypothalamic AMPKα (pAMPKα) and its downstream effector, acetyl-coenzyme A carboxylase alpha (pACCα), compared to controls (Figures 5D and 5F), without changes to total protein levels (Figures 5E and 5F). This phosphoprotein profile is conducive to reduced feeding (López et al., 2008; Minokoshi et al., 2004) and increased activation of BAT in situations such as cold exposure (Figures S4A–4C) or thyroid hormone treatment (López et al., 2010). However, whereas wild-type mice displayed a significant reduction in pAMPKα and pACCα in response to HFD, no such effect was seen in *Bmp8b^−/−^* mice (Figure 5D). This suggested the impairment of central thermogenic regulators in responding to HFD in *Bmp8b^−/−^* mice, mirroring observations from BAT (Figure 3E).

**Figure 6:**
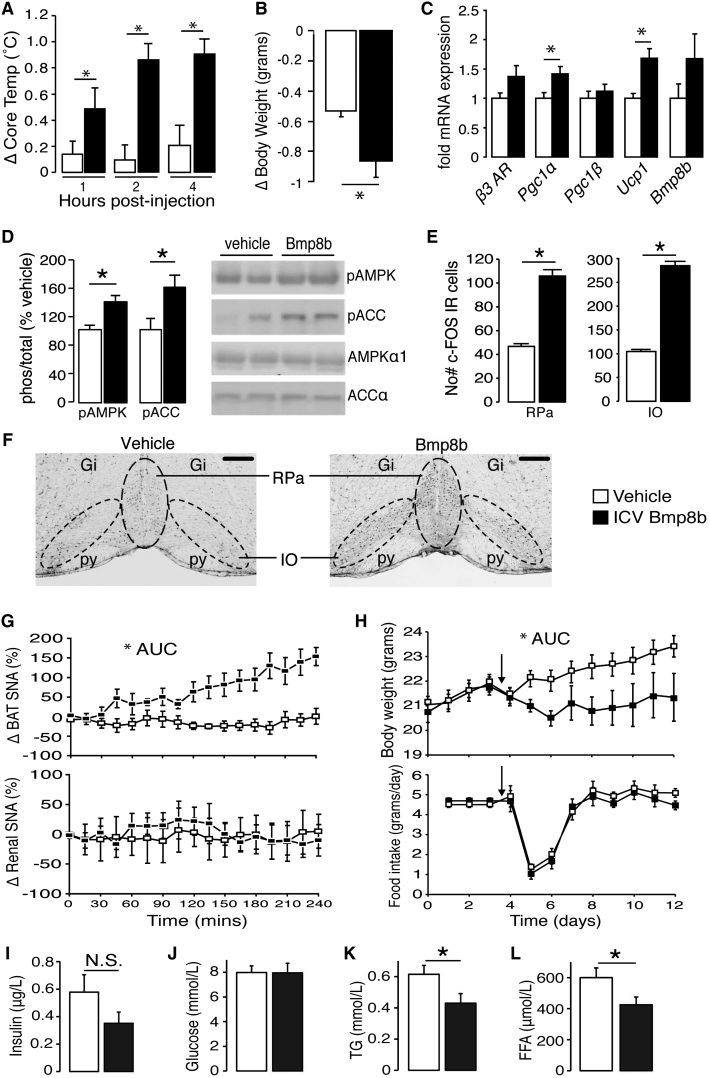
Thermogenic Effect of Central BMP8B Treatment (A) Core body temperature measured by rectal probe following ICV injection with 2 μl of 100 pM BMP8B or vehicle. n = 6–8. (B) Change to body weight of the same animals 4 hr postinjection. n = 6–8. (C) Expression of thermogenic genes in BAT. n = 6–8. (D) One hour post-ICV analysis of pAMPKα and pACCα in the hypothalamus of mice treated with BMP8B or vehicle with representative blots, normalized to β-actin. n = 6–8. (E and F) (E) Numbers of c-FOS immunoreactive (IR) neurons in RPa and inferior olive (IO) under the same conditions with (F) representative sections (Gi, gigantocellular reticular nucleus; IO, inferior olive; py, pyramidal tract; RPa, raphe pallidus; scale bar, 200 μm). n = 5. (G) Change in SNA to BAT and kidney of mice following ICV vehicle or BMP8B, injection at time 0. n = 4–6, age 12–14 weeks. (H–L) (H) Daily body weights of mice treated with chronic BMP8B (100 pM) or vehicle via ICV cannular connected to subcut pump with corresponding food intake and circulating (I) insulin, (J) glucose, (K) triglycerides, and (L) free fatty acids at end of stud. n = 7, age 12 weeks. Error bars indicate SEM. ^∗^p < 0.05 using ANOVA. AUC, area under curve. See also Figure S5.

We next investigated whether intracerebroventricular (ICV) treatment with BMP8B could alter global energy balance by affecting thermogenesis and food intake. Compared to vehicle, BMP8B elicited an increase in core body temperature that lasted for 4 hr (Figure 6A) and was accompanied by increased weight loss (Figure 6B) and induction of thermogenic genes in BAT (Figure 6C). After 1 hr, animals that were treated with BMP8B displayed increased phosphorylation of hypothalamic AMPKα and ACCα (Figure 6D) and increased neuronal activation in nuclei that receive projections from the VMH and coordinate sympathetic outflow to BAT (raphe pallidus nucleus and inferior olive nucleus) (Figures 6E and 6F) (Cannon and Nedergaard, 2004; López et al., 2010). SNA to BAT increased significantly following ICV treatment with BMP8B compared to controls, without changes to renal SNA (Figure 6G).

**Figure S5:**
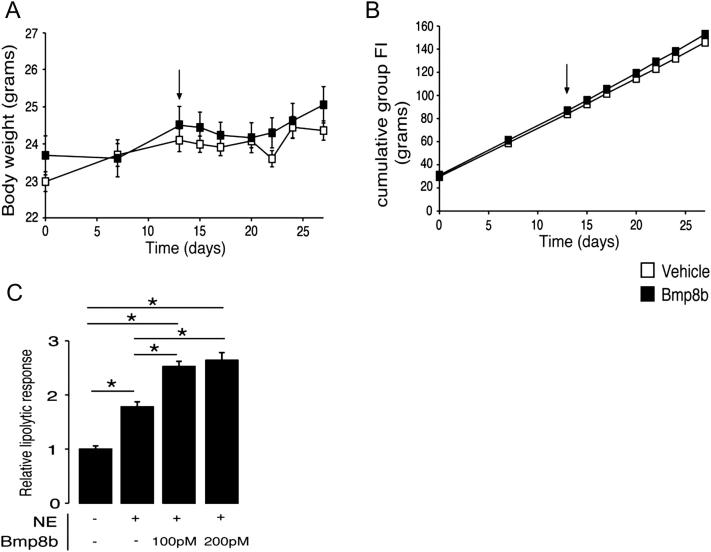
Response to Chronic BMP8B ICV Infusion and Justification of Dose, Related to Figure 6 (A) Body weight and (B) food intake following chronic infusion with vehicle or 100 nM rhBMP8B (0.25 μl/hr) for 2 weeks, via subcutaneous osmotic pumps. Arrow indicates implantation of pumps, n = 7 per group. (C) Dose response in mature brown adipocytes stimulated with 75 nM NE after treatment with differing concentrations of BMP8B using lipolysis as a readout via free glycerol release. n = 3 experiments carried out in triplicate, ^∗^p < 0.05 using ANOVA. Error bars indicate SEM.

In a chronic setting, ICV infusion with BMP8B resulted in marked weight loss, which was maintained during the 8 days of treatment without changes to food intake (Figure 6H). This suggested an increase in energy expenditure. No such response was observed in mice treated peripherally with the same dose (Figures S5A and 5B). ICV BMP8B-treated mice also displayed a tendency for lower serum insulin levels than controls (Figure 6I), along with normal glucose (Figure 6J), lower triglycerides (Figure 6K), and free fatty acids (Figure 6L), indicating potential metabolic benefits of selective BAT activation by BMP8B.

### Central BMP8B's Thermogenic Action Is Dependent on the Level of Activation of Hypothalamic AMPK

**Figure 7:**
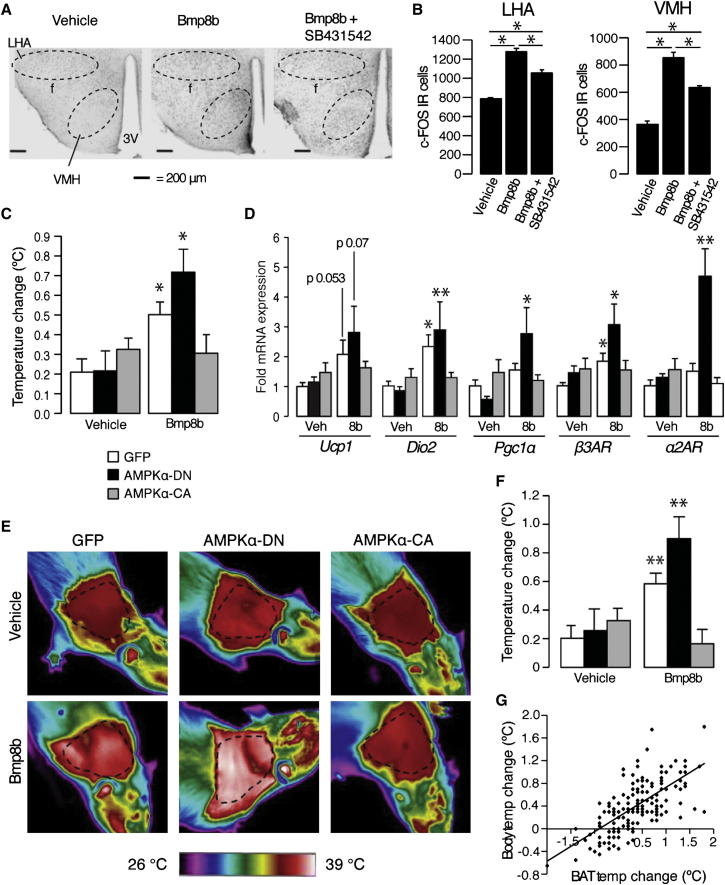
AMPK in the VMH Regulates the Thermogenic Effect of BMP8B (A and B) (A) Representative c-FOS images and (B) total number of c-FOS immunoreactive (IR) neurons in LHA and VMH of mice treated for 2 hr with 2 μl ICV vehicle, 100 pM BMP8B, or BMP8B + 10 uM SB431452. n = 8, age 12 weeks (3V = third ventricle, f = fornix). (C and D) (C) Core body temperature after 2 hr ICV BMP8B treatment in female rats expressing either GFP, DN, or constitutively active (CA) AMPKα in the VMH with (D) mRNA expression in BAT of the same rats, explanted after final treatment. (E–G) (E) Representative thermal images of rats corresponding to the different AMPKα isoforms 2 hr postvehicle or post-BMP8B treatment with (F) spot temperatures adjacent to interscapular BAT depot (dotted line) and (G) correlation between core temperature and BAT temperature increase. n = 8–16. Error bars indicate SEM. Female Sprague-Dawley rats/group, age 10 weeks, mean of four separate 2 hr BMP8B treatments. ^∗^p < 0.05, ^∗∗^p < 0.005, using ANOVA. See also Figure S6.

**Figure S6:**
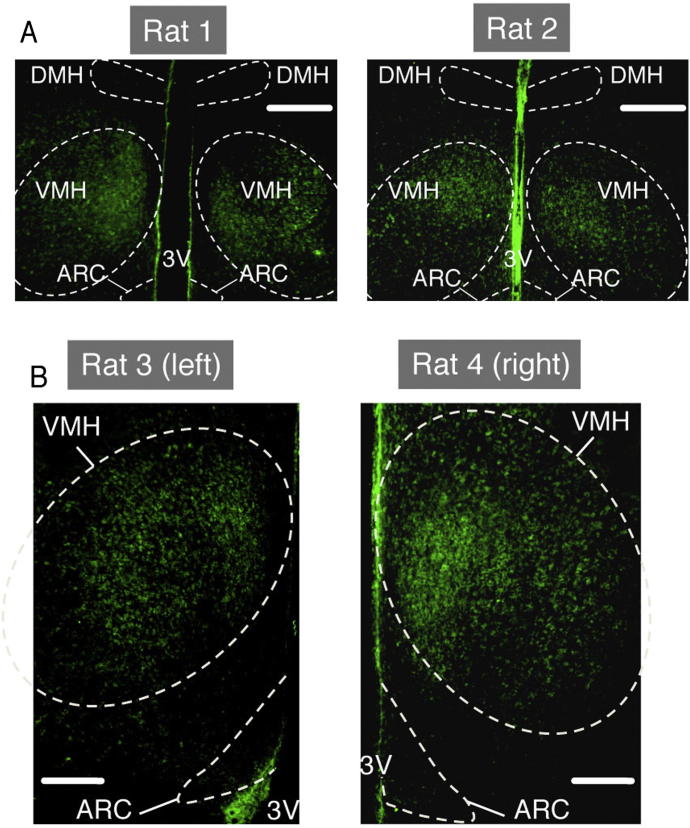
Localization of Stereotaxically Delivered Viral Expression Vectors, Related to Figure 7 (A) Representative brain slices taken from rats injected with GFP-expressing viruses targeted to the VMH as detailed in the experimental procedures, showing VMH localized GFP in relation to that of DMH. (B) Representative brain slices showing GFP expression in VMH in relation to ARC. Intense staining along the apical and basal edges of the 3V is accounted for by increased viral uptake by large numbers of phagocytic supraependymal cells in this region. Slices are taken between Interaural 5.6 mm; Bregma −3.14 mm and Interaural 5.7 mm; Bregma −3.3 mm. 3V = third ventricle, scale bar = 200 μm (panel A), 100 μm (B).

Examination of hypothalamic nuclei following acute ICV treatment with BMP8B revealed increased neuronal activation in the lateral hypothalamic area (LHA) and VMH, which, like BMP8B's effects in BAT, was inhibited by cotreatment with the ALK7 antagonist SB431452 (Figures 7A and 7B). Given the importance of AMPK in the VMH-BAT axis (Cannon and Nedergaard, 2004; López et al., 2010; Martínez de Morentin et al., 2012; Xue et al., 2009), we investigated whether the thermogenic effect of BMP8B was dependent on AMPK activation. Using stereotaxic adenoviral delivery targeted to a large portion of the VMH, AMPKα isoforms carrying either dominant-negative (DN) or constitutively active (CA) mutations were expressed. Immunohistochemistry showed that a small number of neurons in the nearby ARC and dorsomedial nuclei were also infected, but it also showed that the majority of viral expression was restricted to the VMH (Figure S6). The thermogenic effect of BMP8B was increased in the presence of AMPKα-DN expression and was completely ablated by expression of AMPKα-CA when compared to vehicle-treated animals (Figure 7C). Supportive of BAT being the key responder to central BMP8B signaling, levels of BAT thermogenic gene induction mirrored patterns of body temperature change following BMP8B treatment alongside different AMPKα isoforms (Figure 7D). BAT's relative activation following ICV BMP8B was further confirmed by direct measurement using thermal imaging (Figures 7E and 7F) and correlated extremely well with changes in core temperature in a given animal (Figure 7G).

## Discussion

We identify a role for BMP8B as a molecule that is expressed and active in mature BAT and central nervous system (CNS) and that functions to regulate thermogenesis and energy balance. To our knowledge, no previous roles have been described for BMPs in the acute regulation of mature BAT. The coordinated activity of BMP8B in both the hypothalamus and BAT identifies this protein as a fundamental component of energy balance regulation, particularly in response to nutritional changes. BMPs were previously thought to function only in the differentiation of progenitor cells toward a specific cell fate (Tseng et al., 2008; Zhao and Hogan, 1996), but our findings reveal that, at least in the case of BMP8B, their specific signaling abilities can make them potent regulators of mature physiological systems. The selective localization and acute regulation of BMP8B, along with its secretory nature, highlight an attractive mechanism to specifically enhance the activity of endogenous thermogenic machinery.

Our data demonstrate that, physiologically, BMP8B acts as a component of the thermogenic machinery in mature BAT, rather than as a factor driving its formation/differentiation. This concept is supported by the lack of any gross abnormality in BAT morphology in *Bmp8b^−/−^* animals like that seen in animals lacking key brown proadipogenic factors such as PRDM16 or PGC1α (Lin et al., 2004; Seale et al., 2007). Instead, BMP8B “primes” the brown adipocytes' lipolytic machinery to enable a greater thermogenic response in times of increased demand for heat production.

CREB and P38 MAPK are essential for the generation of an appropriate thermogenic response and for Ucp1 induction (Cao et al., 2001; Rim and Kozak, 2002; Sellayah et al., 2011). They have recently been shown to respond to activation of BMP receptor 1A (Sellayah et al., 2011), and we demonstrate that ablation of BMP8B impedes their activation in BAT significantly. In contrast, treating brown adipocytes with BMP8B increased P38 MAPK signaling and increased the pool of active HSL, enabling greater lipolytic activity and likely thermogenic activity in response to a given adrenergic stimulation.

Our studies also support a function of BMP8B in the CNS, where it is expressed in key hypothalamic nuclei controlling energy balance and thermogenesis. Central treatment with BMP8B elicits increased thermogenesis via neuronal activation of regulatory nuclei in the hypothalamus and medulla oblongata (Cannon and Nedergaard, 2004; López et al., 2010) and specific increases in sympathetic tone to BAT. This specificity, along with BMP8B's presence in nuclei such as the VMH, suggests that BMP8B is a bona fide component of central regulation of thermogenesis. The physiological implication of these coordinated peripheral and central functions is such that ablation of BMP8B results in increased propensity for weight gain due to impaired thermogenesis. Following the mild obesogenic challenge of a chow diet, *Bmp8b^−/−^* mice appear to partially compensate by increasing sympathetic activation of BAT to levels above those seen in wild-type mice. However, the underlying impairment is further revealed by challenge with HFD, in which *Bmp8b^−/−^* mice are unable to produce the appropriate thermogenic response.

We hypothesize that, centrally, AMPK acts in opposition to BMP8B to regulate energy expenditure, becoming increasingly active in brain and BAT following treatment with BMP8B. This theory is further supported by our findings in rats with virally induced alterations to AMPKα that are targeted primarily to the VMH, a mechanism we have previously shown to be fundamental to thermogenesis and energy balance (López et al., 2010; Martínez de Morentin et al., 2012). In these studies, localized expression of DN AMPKα results in a greater thermogenic effect of ICV BMP8B, whereas expression of constitutively active AMPKα completely inhibits BMP8B-stimulated activation of BAT. Although viral delivery was focused heavily in the VMH, a small number of cells in the DMH and ARC were also infected, and a contribution of AMPK in these nuclei to BMP8B signaling cannot be entirely ruled out. However, it remains clear that, without the energy-expending effects of BMP8B, the energy-conserving effects of active AMPK are left unchecked in CNS and BAT. This highlights Bmb8b and AMPK as counterregulatory mechanisms that modulate thermogenesis in BAT to control energy balance. Considering that AMPK is already a targetable candidate in peripheral tissues to treat insulin resistance (metformin) (Zhou et al., 2001), the relationship with BMP8B could offer new opportunities for drug design.

On chow diet, the neuropeptide expression profiles suggest increased energy expenditure. Indeed, the detection of positive energy balance may result in the CNS attempting to address this via alterations in the ARC. However, the lack of BMP8B renders this response impotent. The ability of central BMP8B treatment to actively reduce body weight offers an exciting premise for therapeutic applications and demonstrates the importance of understanding thermogenesis at a central regulatory level (Whittle et al., 2011). Targeted upregulation of sympathetic tone to BAT and an increased BAT response to existing activation represent a unique opportunity to design thermogenic antiobesity treatments without the deleterious cardiovascular side effects associated with previous SNS-mediated strategies (Haller and Benowitz, 2000; Torp-Pedersen et al., 2007; Whittle et al., 2011). The fact that BMP8B may do so without a compensatory increase in food intake is further cause to fervently pursue this avenue of research.

## Experimental Procedures

### Materials

Recombinant human BMP8B was purchased from R&D Systems. Antibodies are detailed in the Extended Experimental Procedures. All other chemicals were obtained from Sigma-Aldrich. Diets for animal studies included standard chow (sodium dodecyl sulfate [SDS], 10% calories from lipid) and an HFD (D12451, Research Diets, 45% calories from lipid).

### Cell Culture and Differentiation

The immortalized brown adipocyte cell line was a gift from the laboratory of Johannes Klein and was generated as previously described (Klein et al., 2002). By day 8 postinduction, cells were defined as differentiated if they appeared healthy and lipid replete. T3 and insulin were removed from the media 24 hr prior to any treatment with these molecules. Full details of differentiation and luciferase assays can be found in the Extended Experimental Procedures.

### Lipolysis Assays

Immortalized brown adipocytes were differentiated in 96-well culture plates (seeded 7,000 cells/well). Conditioned media were added with BMP8B and stated antagonists for 2 hr prestimulation. After treatment, media were replaced with serum-free Dulbecco's modified Eagle's medium (DMEM) (still containing any treatments), and cells were stimulated as described. Medium was sampled at the indicated times, and glycerol was measured as an index of lipolysis by using free glycerol reagent (Sigma) against a glycerol standard curve.

### Animals

Unless otherwise stated, all data are from work on females. C57Bl6/J mice were purchased from Charles River. *BMP8b^−/−^* mice were generated as previously described (Zhao et al., 1996) on a C57Bl6/J background and were compared to wild-type littermates. PPARα null mice were obtained from Jackson Laboratory (USA). Sprague-Dawley female rats (9–11 weeks old) were from Central Animal House of the University of Santiago de Compostela (USC). Unless stated, mice and rats were housed in a temperature-controlled room (22°C) with a 12 hr light/dark cycle with free access to diet and water. The UK Home Office and the USC Bioethics Committee approved all animal procedures.

### Diet and Temperature Studies

Standard chow or HFD was administered ad libitum to animals from weaning until indicated. Cold exposure involved single housing of animals, fed standard chow, at 16°C for 1 week, followed by 3 weeks of housing at 4°C. Thermoneutrality involved housing mice at 30°C for 3 weeks. Fasting consisted of removing food for 24 hr, and refeeding involved fasting followed by replacement of food for 24 hr. Fat and lean masses were calculated by time-domain nuclear magnetic resonance (TD-NMR) by using a minispec Live Mice Analyzer LF50 (Bruker).

### NE-Induced Thermogenesis

NE-induced thermogenesis was measured via oxygen consumption by using the *in*direct *ca*lorimeter INCA system (Somedic, Hörby, Sweden) (Alberts et al., 2006). Zirconium oxide sensors were calibrated with reference gases (18% and 25% O_2_ in N_2_) before the experiment. Basal metabolic rate was defined as the last 6 min before injection. The response to NE was the mean of the three highest points after injection minus the basal metabolic rate.

### Sympathetic Nerve Recordings

SNA was measured directly from nerves supplying interscapular BAT and kidney simultaneously, as previously described (Rahmouni et al., 2005). Baseline SNA and hemodynamic variables were recorded for 10 min with rectal temperature maintained at 37.5°C. An average of three separate measurements during the control period was considered to be the baseline value. Integrated voltage after death (background noise) was subtracted from total integrated voltage to calculate real SNA to tissues. Sympathetic nerve responses are expressed as percentage change from baseline.

#### In Response to Cooling

After baseline recordings were taken, the rectal temperature was allowed to fall at a constant and controlled rate, which was the same for each animal (0.25°C/min). SNA to BAT was measured every 2 min.

#### In Response to rhBMP8B

ICV cannulae were implanted 1 week prior to measurements, where rectal temperatures were maintained at 37.5°Cthroughout. Baselines were acquired, and 2 μl rhBMP8B (100 pM) was injected ICV. Measurements were taken every 15 min over 4 hr.

### Stereotaxic Microinjection of Adenoviral Expression Vectors

Rats were placed in a stereotaxic frame (David Kopf Instruments; Tujunga, CA, USA) under ketamine/xylazine anesthesia. The VMH was targeted bilaterally by using a 25 gauge needle (Hamilton; Reno, NV, USA) connected to a 1 μl syringe. The injection was directed to stereotaxic coordinates 2.3/3.3 mm posterior to the bregma (two injections were performed in each VMH), ±0.6 mm lateral to midline and 10.2 mm below the surface of the skull, as described previously (López et al., 2008, 2010; Martínez de Morentin et al., 2012; Varela et al., 2012). Adenoviral vectors (green fluorescent protein [GFP], AMPKα-DN or AMPKα-CA; 1012 pfu/ml [Viraquest; North Liberty, IA, USA]) were delivered at a rate of 200 nl/min for 5 min (1 μl/injection site). GFP control animals were injected each time the stereotaxic frame was used for experimental animals to confirm injection site accuracy (see Figure S6).

### Calculation of Energy Expenditure

Animals were placed in a monitoring system based on their home cages (Ideas Studio) that had the ability to measure oxygen and carbon dioxide concentrations using a system designed by Peter Murgatroyd. Oxygen consumption and carbon dioxide production was measured, and samples were taken at 18 min intervals for a 48 hr period. Energy expenditure was then calculated using indirect calorimetry with the Elia and Livesey constants for respiratory quotient (Elia and Livesey, 1992). Energy expenditure is expressed as Joules/min/mouse by using an adjusted mean bodyweight. This was obtained using analysis of covariance (ANCOVA) with weight as the covariate. ANCOVA is a robust method for comparison of groups with divergent body weight and composition (Arch et al., 2006).

### Statistical Methods

All data are expressed as mean ±SEM. All analyses were performed using the Statistical Package for the Social Sciences (SPSS)/Predictive Analysis Software (PASW) 18.0 and with significance defined as p < 0.05. One-way ANOVA was used for direct comparisons, correcting for multiple variables where applicable. Two-way ANOVA was used to examine interactions between variables. ANCOVA was used to analyze energy expenditure data. Sample sizes and statistical tests used are defined in each figure legend.

Additional information, including oligonucleotide sequences (Tables S3 and S4), is available in the Extended Experimental Procedures.

Extended Experimental Procedures **Antibodies**

Anti-phospho P38MAPK and anti-total P38 MAPK (Promega), anti-phospho CREB, anti-total CREB, anti-phospho MKK3/6, anti-total MKK3/6, anti-pHSL, anti-pAMPK (Cell Signaling Technology), anti-beta actin (AbCam). ACC, pACC-Ser^79^, AMPKα1, AMPKα2 (*Upstate;* Temecula, CA, USA); FAS (*BD*, Franklin Lakes, NJ, USA), pAMPK-Thr^172^ (*Cell Signaling;* Danvers; MA, USA). Immobilon-P membranes were from Millipore.

**Cell Culture**

C57 BAT or HIB 1B Cells were maintained in DMEM with 10% fetal bovine serum (FBS), 20 mM L-glutamine, 100 units/ml Penicillin and 100 μg/ml Streptomycin at 37°C in 5% CO_2_. To differentiate, cells were plated in 24 well plates (30,000 cells/well) and T3 and insulin were added to the media at concentrations of 1 nM and 20 nM respectively until 75%–80% confluence was reached. At this point IBMX (500 μM), dexamethasone (2 mM) and indomethacin (125 μM) were added to the media overnight to induce differentiation, before changing back to the growth media with just T3 and insulin added which was changed every 48 hr.

**Luciferase Reporter Assay**

HEK293 cells or HIB-1 brown fat cell line cells were cultured in 96-well culture plates and transfected using Lipofectamine (Invitrogen) with 250 ng either pGL3 basic (Promega), or pGL3 containing 3 Kb of the proximal BMP8B promoter upstream of the luciferase gene. Each set of cells was co-transfected with pCDNA 3.1 (Invitrogen) empty vector or increasing amounts of pCDNA 3.1 containing either thyroid hormone receptor alpha (THRα), beta 1 (THRβ1) or beta 2 (THRβ2), or expression vectors for PPARα (pSG5- PPARα) and PPARγ (pSG5-PPARγ). 2 ng of renilla luciferase plasmid (pTKRL, Promega) was used as control for transfection efficiency. When indicated, cells were treated with 1μ GW7647, 10μ rosiglitazone or 1 mM dibutyril cAMP. Luciferase activity was assayed 24 hr later using Stop and Glo reagent (Promega) and a Centro 960 microplate luminometer (Berthold). Luciferase activity values from wells containing no BMP8B promoter fragment were subtracted from the corresponding experimental well values to correct for background. All values were normalized to renilla luciferase activity.

**Serum Biochemistry**

Serum biochemicals were measured using the following commercially available kits: Triglycerides, cholesterol, HDL and Glucose were measured enzymatically (Dade-Behring). LDL was calculated using the Friedwald formula (LDL = Cholesterol - HDL - (Triglycerides/2.2). NEFA were measured by enzymatic assay (Roche). Insulin and leptin were measured using electrochemical luminescence immunoassay (MesoScale-Discovery). 3-OH butyrate was measured using a colorimetric assay (Inverness Biomedical). T4 was measured using time resolved fluoroimmunoassay (DELFIA) (Perkin-Elmer). Corticosterone was measured using an immunoassay (IDS).

**Glucose and Insulin Tolerance Tests**

For glucose tolerance test (GTT), mice were fasted overnight before basal measurement of blood glucose, followed by intraperitoneal (IP) injection with glucose (1 g/kg). Blood glucose was measured using a one-touch ultra glucose meter (lifescan) at 10, 20, 30, 60, and 120 min postinjection. For Insulin tolerance test (ITT) mice were fasted for 4 hr from 8 am before basal measurement of blood glucose. Mice were then injected IP with 0.75 U/kg of insulin and blood glucose measured as for GTT.

**NE-Induced Thermogenesis**

Eight individually housed mice were monitored simultaneously and airflow was 1 l/min. Mice housed at varying temperatures were anaesthetized with pentobarbital (45 mg/kg i.p.). The mice were measured at 33°C to obtain basal values during 30 min. After basal readings the mice were removed from the metabolic chambers for a short time (6 min) and injected subcutaneously with NE (1 mg/kg) before return to the metabolic chambers for 60 min.

**T3 + T4 Induced Hyperthyroidism**

12-week-old C57Bl6/J mice were implanted dorsally with subcutaneous osmotic pumps (Alzet) containing either 10 mM NaOH (vehicle), a dose of 5 μg T4 + 2 μg T3/100 g/day (mild) or 20 μg T4 + 8 μg T3/100 g/day (intensive) over a 10 day period. Body weights were monitored throughout and at the end of the study tissues were explanted and processed as described.

**Tissue Collection and Histology**

All animal tissues for protein or RNA extraction were frozen at time of collection unless otherwise stated and later ground to a fine powder using a sterile pestle and mortar on liquid nitrogen. Samples for histology were placed in 10% buffered formalin overnight before transfer to 70% ethanol and later embedding in paraffin. Multiple sections were stained with hematoxylin and eosin for morphological analysis. Lipid droplet size and number of nuclei were assessed visually, whereby three individuals analyzed two images from each animal's BAT sections and scored them 1–5 blinded; the results were averaged. BAT for mature adipocyte and stromal-vascular fractionation was excised from 6-week-old female mice into DMEM before mincing with sterile surgical scissors and digestion at 37°C with shaking for ∼45 min (10 ml Hanks buffered salt solution, 2% BSA, 2 mg/ml Type 2 collagenase). Digests were centrifuged at 700 g for 10 min and the floating mature adipocytes and pelleted stromal-vascular cells each removed into Buffer RLT (QIAGEN) with 20 μl/ml 2-mercaptoethanol for subsequent isolation of RNA.

**Western Blot Analysis**

Powdered tissue or collected cultured cells were resuspended in lysis buffer (20 mM Tris-HCL, 150 mM NaCl, 1 mM EGTA, 1 mM EDTA, 1% Triton X-100, 1 mM vanadate, pH7.5) with added protease and phosphatase inhibitor cocktails according to manufacturers instruction (Sigma). After lysis, lysates were cleared by centrifugation at 10,000 g for 10 min at 4°C. Protein concentrations of the supernatants were determined by D_c_ Protein assay (Bio-Rad). Proteins were diluted in Laemmli buffer with 2-mercaptoethanol. 20 ng of proteins were separated by SDS-polyacrylamide gel (10%) electrophoresis and transferred to Immobilon-P membrane. Membranes were blocked for 1 hr at room temperatures and incubated overnight at 4°C with the indicated antibody. Bound primary antibodies where detected using peroxidase-coupled secondary antibodies and enhanced chemiluminescence (Amersham). Relative quantification of band densitometry was calculated by digitally photographing exposed films and using Genesnap and Genetools software (Syngene).

**Q-RT-PCR**

Total RNA was isolated from cells and BAT fractions using Buffer RLT and purified by RNeasy Mini columns (QIAGEN). RNA was isolated from ground tissues using STAT-60 reagent (TEL-TEST) followed by chloroform extraction and isopropanol precipitation. Complimentary DNA was generated from 500 ng of RNA using M-MLV reverse transcriptase and master mix (Promega) in a 20 μl reaction with 2.5 mM MgCl_2_, 1.25 mM dNTPs and 5 μg/ml random hexamers at 37°C for 1 hr. cDNA was diluted 75 fold and 5 μl of diluted cDNA was used in a 12 μl real time PCR reaction using TaqMan primers and probes or SYBR green reagent (Applied Biosystems) according to manufacturer's instructions. Reactions were run in duplicate for each sample and quantified in the ABI Prism 7900 sequence detection system (Applied Biosystems). Data expressed as arbitrary units and expression of target genes corrected to the geometric average of four housekeeping genes: 18S, β2-microglobulin, β-actin and 36B4 using Bestkeeper (freeware). Sequences of primers and probes used are listed in Table S3.

**Temperature Measurements**

Body temperature was recorded with a rectal probe connected to digital thermometer (BAT-12 Microprobe-Thermometer; Physitemp; NJ, US). Skin temperature surrounding BAT was recorded with an infrared camera (E60bx: Compact-Infrared-Thermal-Imaging-Camera; FLIR; West Malling, Kent, UK) and analyzed with a specific software package (FLIR-Tools-Software; FLIR; West Malling, Kent, UK). We used 8–16 female rats per group and for each animal 3 to 4 pictures were analyzed. The skin temperature surrounding BAT for one particular animal was calculated as the average temperature recorded by analyzing those pictures.

**In Situ Hybridization**

Coronal hypothalamic sections (16 μm) were cut on a cryostat and immediately stored at −80°C until hybridization. Specific oligos for AgRP, BMP8B, CART, NPY and POMC and TRH detection were used (Table S4). These probes were 3′ end labeled with ^35^S-αdATP using terminal deoxynucleotidyl transferase (Amersham Biosciences, UK). In situ hybridizations were performed as previously published (Lage et al., 2010; Lopez et al., 2006, 2008). Between seven and ten animals per experimental group were used. We used between 10 and 16 sections for each animal. The mean of these 10–16 values was used as the densitometry value for each animal.

**c-FOS Immunohistochemistry**

Diaminobenzidine (DAB) immunohistochemistry (*Dako EnVision*), was performed as described previously (López et al., 2006, 2008, 2010). Using a rabbit anti-c-FOS (*Santa Cruz Biotechnology*). Mice brains were fixed and sectioned at 50 μm by using a vibratome. Free-floating sections were consecutively incubated in: 1) anti-c-FOS antibody (Santa Cruz) at a dilution of 1:1000, overnight at 4°C; 2) 3% hydrogen peroxide (Merck) for 10 min to block endogenous peroxidase; 3) detection system (Envision HRP anti-rabbit, Dakocytomation) for 30 min and 4) 3,3′-diaminobenzidine tetrahydrochloride (Dakocytomation) for 10 min. Between steps, sections were washed twice for 10 min with TBS (0.05 M Tris buffer of pH 7.6 containing 0.3 M NaCl) and after step 4 with distilled water. All dilutions were made in TBS.

Sections were analyzed qualitatively with an Olympus light microscope, and c-FOS-immunoreactivity (IR) in appropriate nuclei (raphe pallidus, RPa and inferior olive, IO), lateral hypothalamic area (LHA) or ventromedial hypothalamic nucleus (VMH) was quantified. For each region, ten to twenty adjacent sections were counted and for each section (for the IO) both sides of the brain were counted. The average number of stained cells on both sides was used to represent the number of c-FOS-IR-positive cells in that section. Animals from each treatment group (n = 5) were compared by using the mean number of stained cells in all sections counted in that nucleus.

## Supplemental Information

Document S1. Tables S1–S4

Document S2. Article plus Supplemental Information
