# Supplementary material for: BMP8B Increases Brown Adipose Tissue Thermogenesis through Both Central and Peripheral Actions
Source: Cell. 2012 May 11;149(4):871–85. doi: 10.1016/j.cell.2012.02.066 (PMC3383997; doi:10.1016/j.cell.2012.02.066)
Supplement: Document S1. Tables S1–S4 [file mmc1.pdf]

Table S1. Temperature-Dependant Ratios of *Bmp8b*$^{-/-}$ Pups, Related to Figure 2

**20°C**			**23°C**		
Genotype	*Observed*	*Expected*	Genotype	*Observed*	*Expected*
(+/+)	142 (30%)	120	(+/+)	283 (28%)	252
(+/-)	283 (59%)	239	(+/-)	529 (53%)	502
(-/-)	54 (**11%**)	120	(-/-)	194 (**19%**)	252
Total	479	479	Total	1006	1006

$Chi^2 = 48.4$ $P < 0.0001$ $Chi^2 = 18.6$ $P < 0.0001$

Total numbers of pups of different genotypes born over the course of two years when ambient housing temperature was kept at the indicated temperature. Breeding was set up using mice heterozygous for the Bmp8b deletion. Percentages for that year indicated in brackets.

Table S2. Serum Biochemistry of *Bmp8b*$^{-/-}$ Mice, Related to Figure 2

		Chow		HFD		
**Marker**	**Units**	***Bmp8b*** $^{+/+}$	***Bmp8b*** $^{-/-}$	***Bmp8b*** $^{+/+}$	***Bmp8b*** $^{-/-}$	**Sig.**
Cholesterol	mmol/L	1.85 (+/-) 0.13	1.79 (+/-) 0.15	3.83 (+/-) 0.14	4.22 (+/-) 0.20	b
Triglycerides	mmol/L	0.94 (+/-) 0.08	0.92 (+/-) 0.07	0.72 (+/-) 0.07	0.60 (+/-) 0.04	b
HDL	mmol/L	0.94 (+/-) 0.13	0.83 (+/-) 0.17	1.55 (+/-) 0.06	1.54 (+/-) 0.11	b
LDL	mmol/L	0.50 (+/-) 0.10	0.53 (+/-) 0.16	1.96 (+/-) 0.13	2.40 (+/-) 0.18	b
Glucose	mmol/L	11.29 (+/-) 0.59	10.68 (+/-) 0.46	12.74 (+/-) 0.45	13.20 (+/-) 0.59	
NEFA	µmol/L	1151(+/-) 94	1238 (+/-) 103	1048 (+/-) 104	949 (+/-) 104	
Insulin	µg/L	0.47 (+/-) 0.13	0.54 (+/-) 0.10	1.23(+/-) 0.24	1.78 (+/-) 0.30	b
Leptin	pg/ml	2813 (+/-) 399	4518 (+/-) 973	51029 (+/-) 10954	79060 (+/-) 9890	b
Corticosterone	ng/ml	112.3 (+/-) 31.6	68.7 (+/-) 21.2	140.8 (+/-) 28.9	153.5 (+/-) 14.8	
3-OH Butyrate	µmol/L	278.4 (+/-) 65.6	349.1 (+/-) 58.8	355.7 (+/-) 81.1	431.8 (+/-) 47.2	
T4	nmol/L	61.35 (+/-) 6.09	49.03 (+/-) 8.23	61.50 (+/-) 2.55	54.92 (+/-) 3.29	

Serum biochemistry analysis of levels of a range of metabolically relevant molecules in WT and KO mice fed standard chow and HFD. N = 7 per group, aged 20 weeks. Annotation indicates significant effect of a = genotype, b = diet, c = significant genotype-diet interaction where $p < 0.05$ using 2-Way ANOVA.

Table S3. List of Oligonucleotide Sequences, Related to Experimental Procedures

Gene	Forward Primer	Reverse Primer	Probe/SYBR green
MOUSE			
18s	CGGCTACCACATCCAAGGAA	GTCGGAATTACCGCGGCT	GAGGGCAAGTCTGGTGCCAG
Actb	GCTCTGGCTCCTAGCACCAT	GCCACCGATCCACACAGAGT	ATCAAGATCATTGCTCCTCCTGAGCGC
Adra2a	CGCAGGCCATCGAGTACAA	GGTGACAATGATGGCCTTGAT	SYBR
Adrb3	CCAGCCAGCCCTGTTGA	GGACGCGCACCTTCATAGC	SYBR
Arbp (36B4)	TCTCCAGAGGCACCATTGAAA	CTCGCTGGCTCCCACCTT	TCTGAGTGATGTGCAGCTGATAAAGACTG GA
B2m	GGTCTTTCTCTGGTGCTTGTC	GTATGTTCGGCTTCCCATTC	SYBR
Bmp2	AACACCGTGCGCAGCTT	GCCGTTTTCCCACTCATCTC	SYBR
Bmp4	GCCATTCCGTAGTGCCATT	TTGCTGGAAAGGCTCAGAGA	SYBR
Bmp5	CAAGAAGCACGAACTCTATGTGAGT	CCCTTCTGGTGCTATGATCCA	SYBR
Bmp6	CGCAGGACAGCGCTTTC	GTCGTACTCCACCAGGTTCACA	SYBR
Bmp7	TGGATGGGCAGAGCATCAA	GGTCCATGCCGTCCAATC	SYBR
Bmp8a	AACCATGCCATCTTGCAGTCT	CAGAGGTGGCACTCAGTTTGG	TGTCCCCAAGGCATGCTGTGCA
Bmp8b	TCCACCAACCACGCCACTAT	CAGTAGGCACACAGCACACCT	CAGGCCCTGGTACATCTGATGAAGCC
Cd36	GCCAAGCTATTGCGACATGA	TCTCAATGTCCGAGACTTTTCAAC	CACAGACGCAGCCTCCTTTCCACCT
Cpt1b	GCGTGCCAGCCACAATTC	TCCATGCGGTAATATGCTTCAT	CCGGTACTTGGATTCTGTGCGGCC
Dio2	TGCGCTGTGTCTGGAACAG	CTGGAATTGGGAGCATCTTCA	SYBR
Elovl3	ATGAATTTCTCACGCGGGTTA	GAGCTTACCCAGTACTCCTCCAAA	SYBR
Fas	GCCCAGACAGAGAAGAGGCA	CTGACTCGGGCAACTTCCC	GGAGGAGGTGGTGATAGCCGGTATGTC
Hsl	GGAGCACTACAAACGCAACGA	TCGGCCACCGGTAAAGAG	CAGGCCTCAGTGTGACCGCCAGTT
Lcad	GCATGAAACCAAACGTCTGGA	TGTTTTGTAATTCAGATGCCCAGT	TCCGGTTCTGCTTCCATGGCAAAA
Pgc1a	AACCACACCCACAGGATCAGA	CTCTTCGCTTTATTGCTCCATGA	CAAACCCTGCCATTGTTAAGACCGAGAA
Pgc1b	GGCCTTGTGTCAAGGTGGAT	GGTGCTTATGCAGTTCCGTACA	AGACCCCCACACTGCGGGCTC
Ppara	CCTCAGGGTACCACTAGGGAGT	GCCCGAATAGTTCGCCGAAA	CACGCATGTGAAGGCTGTAAGCGCTT
Pparg1	TTTAAAAACAAGACTACCCTTTACTGAAATT	AGAGGTCCACAGAGCTGATTCC	AGAGATGCCATTCTGGCCCACCAACTT
Pparg2	GATGCACTGCCTATGAGCACTT	AGAGGTCCACAGAGCTGATTCC	AGAGATGCCATTCTGGCCCACCAACTT
Prkaca	TCCTTTGGGCGAGTGATGC	GCGAAGAAGGGTGGGTAAC	SYBR
Prkar1a	CTTTAGCCAAGGCCATCGAA	AATGTCACTTCTCTCGTTATCAT	SYBR
Prkar2a	AGAGTGACTCGGACTCGGAAG	GGTCAGTATGACAACCGTGGC	SYBR
Srebp1c	GCCATGGATTGCACATTTGA	GGCCCGGGAAGTCACTG	GACATGCTCCAGCTCATCAACAACCAAG
Ucp1	CCCGCTGGACACTGCC	ACCTAATGGTACTGGAAGCCTGG	AAGTCCGCCTTCAGATCCAAGGTGAAG
RAT			
18s	CGGCTACCACATCCAAGGAA	GTCGGAATTACCGCGGCT	GAGGGCAAGTCTGGTGCCAG
Adrα2a	GGTGGTGATCGGCGTGTT	CGACGGCTATGAGCGTGTAG	SYBR
Adrβ3	CCTTCCCAGCTAGCCCTGTT	TGCTAGATCTCCATGGTCCTTCA	SYBR
Dio2	ACGGAGGCAAACTGGTCCAT	CACTTAGTTCCCGTGTAAGCGTAGTC	SYBR
Hprt	AGCCGACCGGTTCTGTCAT	GGTCATAACCTGGTTCATCATCAC	SYBR
Pgc1α	CGATCACCATATTCCAGGTCAAG	CGATGTGTGCGGTGTCTGTAGT	SYBR
Ucp1	CAATGACCATGTACACCAAGGAA	GATCCGAGTCGCAGAAAAGAA	SYBR

Comprehensive list of primers and probes used for TaqMan analysis of gene expression. “SYBR” indicates no probe was required as SYBR Green reagent was used to quantify amplicon levels.

Table S4. Antisense Oligonucleotides for In Situ Hybridisation Analysis, Related to Experimental Procedures

mRNA	Genbank accession number	Sequence
**AgRP**	NM_007427	5’ – CGACGCGGAGAACGAGACTCGCGGTTCTGTGGATCTAGCACCTCTGCC – 3’
**Bmp8b**	NM_007559	5’ – GAAATGGCACTCAGCTCAGTAGGCACAGCACACCTTGGG – 3’
**CART**	NM_013732	5’ – ACAGTCACACAGCTTCCCGATCCTGGCCCCTTTC – 3’
**NPY**	AF273768	5’ – GGGCGTTTTCTGTGCTTTCCTTCATTAAGAGGTCTG – 3’
**POMC**	NM_008895	5’ – CTTGATGATGGCGTTCTTGAAGAGCGTCACCAGGGGCGTCTGGCTCTT – 3’

List of all hybridisation probes with accession numbers of the mRNA template used to generate them.
